# Synthesis and Structure–Property Relationship of Amphiphilic Poly(2-ethyl-*co*-2-(alkyl/aryl)-2-oxazoline) Copolymers

**DOI:** 10.1021/acsomega.2c04809

**Published:** 2022-10-26

**Authors:** Taha Behroozi Kohlan, Asu Ece Atespare, Mehmet Yildiz, Yusuf Ziya Menceloglu, Serkan Unal, Bekir Dizman

**Affiliations:** † Integrated Manufacturing Technologies Research and Application Center & Composite Technologies Center of Excellence, Sabanci University, Istanbul 34956, Turkey; ‡ Faculty of Engineering and Natural Sciences, Materials Science and Nano Engineering, Sabanci University, Istanbul 34956, Turkey

## Abstract

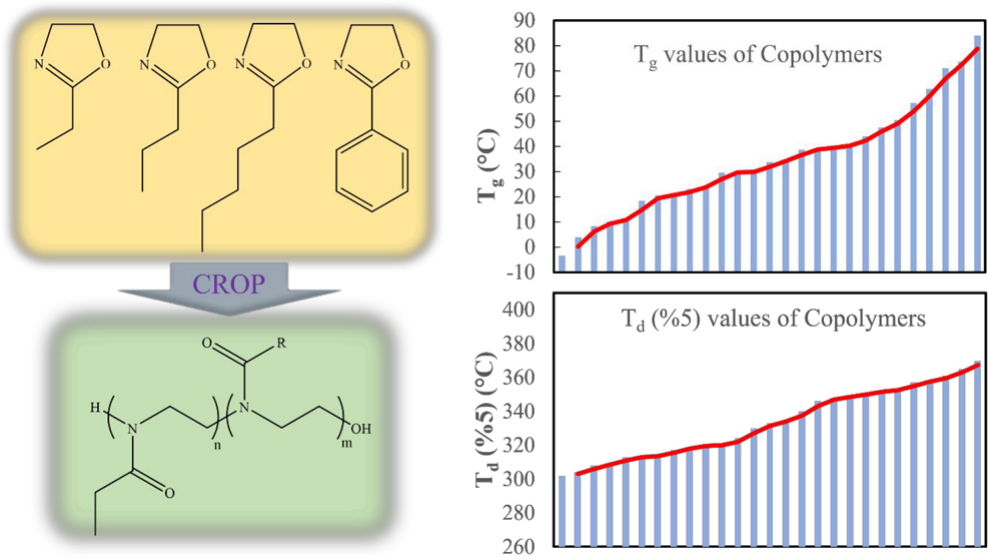

Poly(2-oxazoline)s (POZs) are widely investigated for their applications in various fields due to their unique properties. To exploit and combine different characteristics of the POZ family, 2-oxazoline monomers can be copolymerized to prepare tailor-made copolymers with the desired glass transition temperature (*T*_g_), melting temperature (*T*_m_), amphiphilicity, and functionality. Here, we report the synthesis and characterization of 2-oxazoline monomers and a range of POZ copolymers produced, thereof. 2-Propyl-2-oxazoline (PrOZ) and 2-pentyl-2-oxazoline (PeOZ) monomers were synthesized by two different methods starting from nitriles or carboxylic acids. A number of POZ copolymers were synthesized by copolymerization of 2-ethyl-2-oxazoline (EOZ) with either one of PrOZ, PeOZ, or 2-phenyl-2-oxazoline (PhOZ) at three different compositions (25:75, 50:50, and 75:25) and three molecular weights (1000, 2000, and 5000 Da). The successful synthesis of the monomers and copolymers was demonstrated through their structural analysis by ^1^H NMR and FTIR. SEC results confirmed the targeted molar masses of the copolymers and living nature of the polymerization by showing low dispersity values. Thermal properties of the copolymers were studied using DSC and TGA. DSC studies revealed the amorph and random state of the copolymers with obtained *T*_g_ values for the copolymers in the range of −3 to 84 °C depending on their molecular weight and type of the side chain. While the presence of longer aliphatic side chains resulted in lower *T*_g_ values, the presence of 2-phenyl substituents on the polymer led to higher *T*_g_ values. The decomposition temperatures determined by TGA were in the range of 328 to 383 °C depending on the molecular weight, composition, and side chain of the copolymers. It was observed that higher molecular weights led to higher *T*_g_ values and decomposition temperatures. While copolymers with aliphatic side chains exhibited a single-step decomposition profile, the decomposition of copolymers having aromatic side chains occurred in multiple steps. The variations in the molecular weight, composition, and side chains of the copolymers resulted in a library of tailorable amphiphilic copolymers suitable for multiple applications ranging from biomedical applications to composite manufacturing.

## 1 Introduction

Discovered more than 50 years ago, poly(2-oxazoline)s (POZs) are of great interest for the scientific community owing to their unique properties including ease of synthesis, tailorable properties, versatile functionalities, stimuli-responsiveness, and biocompatibility.^1,2^ POZs are obtained through cationic ring-opening polymerization (CROP) of 2-oxazoline monomers, which are 5-membered cyclic imino-ethers.^3,4^ CROP is a class of chain growth polymerization. The polymerization is initiated by the addition of an electrophilic initiator to the monomer solution. In the initiation step of 2-oxazolines, a nucleophilic attack of the nitrogen lone pair results in the formation of an oxazolinium cation.^5^ The polymerization proceeds with the addition of monomers to the active site of the growing chain and is terminated by addition of a nucleophile to the polymerization medium.^6^ The polymerization of 2-oxazolines is driven by the isomerization of cyclic imino-ethers to more stable tertiary amides which are thermodynamically more favorable.^7^

The unique chemistry of POZs offers the possibility to tailor the polymer structure to obtain desired properties by varying the initiators, 2-substituent groups of the monomer, and the terminating agents.^8^ Even though the use of numerous initiators and terminating agents has been reported in the literature to fine-tune the chemical and physical properties of POZs, altering the 2-substituent group of the monomer remains the most prevalent method for adjusting the polymer properties due to the large number of side chains as opposed to two terminal groups brought by initiator and terminating agent. Pendant side groups of the POZs greatly affect various properties of the polymer including solubility in water and organic solvents, crystallinity, stimuli-responsiveness, and amphiphilicity.^9,10^ In recent years, POZs with various pendant groups including alkyl, aryl,^11^ amino,^12^ aldehyde,^13^ alkene,^14^ and alkynes^15,16^ giving rise to different properties have been investigated for various applications including drug delivery,^15,17,18^ coatings,^19,20^ and composites.^21^

Since CROP of 2-oxazolines is a heat-induced polymerization, two main methods of heat transfer, namely, microwave irradiation and conventional heating, have been proposed and investigated to control the polymerization rate.^22^ The scale of the synthesis, targeted molecular weight, and the synthesis infrastructure are important parameters for choosing the heating method. Despite the advantages of microwave assisted synthesis of the POZs such as shorter polymerization time and overcoming the limitations regarding the boiling points of the reactants through pressurized reactions,^23^ there are limitations in the industrial use of this method. The limited penetration depth of the irradiation, increased heat loss in larger systems,^24^ and the obstacles of incorporating the microwave assisted systems to the existing plants in terms of the required investments limit the possibility of using microwave assisted synthesis systems for large scale POZ production.^25^

The hydrophilicity along with other properties of POZs can be fine-tuned by altering the side chain of the utilized monomer. The amide group on the polymer backbone provides an intrinsic hydrophilicity to the polymer, whereas the nature of 2-substituent groups can make the polymer hydrophilic, hydrophobic, or amphiphilic. For instance, the presence of methyl and ethyl pendant groups makes the polymers hydrophilic, whereas the presence of phenyl and pentyl pendant groups results in hydrophobic polymers.^26^ Moreover, the combination of monomers with varying degrees of hydrophilicity and hydrophobicity provides an amphiphilic character to the polymers. Therefore, the copolymerization of different monomers at varying compositions can be used as an effective mean to obtain POZ polymers with favorable properties such as amphiphilicity, and thermal and mechanical properties, etc. In addition to the types of monomers and compositions of the copolymers, the control of molecular weights of polymers substantially affects these properties.^27^ Copolymerization of 2-oxazoline monomers is feasible due to the controlled or living nature of the polymerization, which can be used to synthesize random,^28^ gradient,^29^ and block copolymers.^30^

Although the POZs are widely used for biomedical applications with an emphasis on solution properties and thermoresponsiveness, other application fields such as organic electronics^31^ and carbon-based nanomaterial modifications^32^ have been reported owing to their fine-tunable properties. For such applications, the properties of POZs in the solid state such as *T*_g_, crystallinity, and thermal degradation are of great importance. Low molecular weight polymers are being widely investigated for their potential applications in additive manufacturing, injection molding, and plasticizing.^33,34^ Moreover, these polymers can be used as compatibilizers to enhance the processability of materials.^35^ Rubberlike materials such as polymers that exhibit a *T*_g_ below their utilization temperature are used in a variety of fields including adhesives, compatibilizers, plasticizers, fillers, etc.^36^ On the other hand, crystallinity of a polymer contributes to its application in other fields such as encapsulation, entrapment, and thermoresponsive release of desired substances.^37^ Thus, the ability to tailor the polymer structure in such a way to exhibit the desired thermal properties is an important factor in its applications in various fields. It has been reported that the functionality of the side chain, length of the side chains in the case of an aliphatic species, and the number of repeating units greatly affect the thermal properties of POZs.^38^ For instance, side chains up to three carbons result in amorphous polymers while poly(2-butyl-2-oxazoline) shows a semicrystalline behavior with a melting temperature of 150 °C.^39^ Moreover, thermal degradation temperature defines the maximum temperature, conditions of polymer processing and production method, and the final application of the polymer.^40^ Thus, we sought to investigate the properties of low molar mass POZ copolymers to examine the possibility of exploiting their properties and their implementation in novel applications.

In this study, we report the synthesis of two 2-oxazoline monomers (PrOZ and PeOZ) via two methods and a scalable synthesis method via conventional heating to obtain a library of well-defined POZ copolymers having different molecular weights and compositions. The synthesized PrOZ and PeOZ and EOZ monomers had aliphatic groups, whereas PhOZ contained an aromatic group on the 2-position. EOZ was copolymerized with other monomers to obtain PEOZ–PPrOZ, PEOZ–PPeOZ, and PEOZ–PPhOZ copolymers at three different compositions (25:75, 50:50, and 75:25) and three different relatively low molar masses (1000, 2000, and 5000 g/mol). The obtained POZ copolymers were characterized by ^1^H NMR, FTIR, and SEC to study their structure, copolymerization efficiency at the implemented reaction conditions, molecular weights, and dispersity indices. Thermal properties such as crystallinity, *T*_g_, and thermal degradation were also widely investigated by DSC and TGA to study the effect of the side chain type and length along with the number of repeating units on the polymer properties.

## 2 Experimental Section

### 2.1 Materials

2-Ethyl-2-oxazoline (EOZ), 2-phenyl-2-oxazoline (PhOZ), chlorobenzene, methanol, and hexanoic acid were purchased from Sigma-Aldrich. Monomers and chlorobenzene were dried over calcium hydride (CaH_2_), distilled, and kept over 4 Å molecular sieves before their use in polymerizations. Trifluoromethanesulfonic acid (TfOH) and oxalyl chloride were purchased from Acros Organics. Butyronitrile and 2-chloroethylamine hydrochloride were purchased from ABCR. Sodium hydroxide (NaOH) and potassium hydroxide (KOH) were purchased from ISOLAB. Ethanolamine, CaH_2,_ dichloromethane (DCM), zinc acetate dihydrate (Zn(OAc)_2_·2H_2_O), triethylamine (TEA), and sodium sulfate (Na_2_SO_4_) were purchased from Merck. Unless it is stated otherwise, all chemicals were used as received.

### 2.2 Instruments

^1^H NMR spectra were recorded on a 500 MHz Varian spectrometer in CDCl_3_ and CD_3_OD. FTIR spectra were recorded on ThermoScientific Nicolet iS50 FTIR spectrometer using an attenuated total reflectance (ATR) accessory. The transmission mode was used, and the resolution was 16 cm^–1^. Size exclusion chromatography (SEC) measurements were carried out on Malvern VISCOTEK GPCmax-Viscotek TDA305 with mixed D5000-D3000-D1000-DGuard column and refractive index detector. The column temperature was 55 °C, and the injection volume was 100 μL. DMF was used as an eluent at a flow rate of 0.7 mL/min, and molecular weights were calculated using both poly(methyl methacrylate) and poly(2-ethyl-2-oxazoline) (PEOZ) standards. PEOZ standards were prepared by calibrating the instrument with five PEOZ polymers with the *M*_p_ values of 500, 1000, 2000, 5000, and 10000 Da synthesized with the same procedure as described in the General Polymer Synthesis Procedure section. The absolute molecular weights of PEOZ standards were obtained from MALDI-TOF (Bruker microflex LT MALDI-TOF MS using (α-cyano-4-hydroxycinnamic acid) and *trans*-2-[3-(4-*tert*-butyl-phenyl)-2-methyl-2-propenylidene] malononitrile (DCTB); sodium trifluoroacetate; and sodium iodide solution) prior to use. Differential scanning calorimetry (DSC) measurements were carried out using Mettler Toledo DSC 3+ instrument. DSC thermograms were obtained from the second heating run at 10 °C/min, after the first run of heating up to 250 °C and cooling down to −50 °C at 10 °C/min, under nitrogen atmosphere, in order to determine the *T*_g_ values. TGA measurements were performed on a Mettler Toledo TGA/DSC 3+ in the range 25–700 °C. Samples of 10–100 mg were heated at 10 °C min^–1^ under nitrogen atmosphere with a flow rate of 100 mL min^–1^. The samples were dried at 110 °C for 30 min prior to analysis to remove any moisture or residual solvent. The onset temperatures of thermal degradation are obtained where the tangent line at the inflection point of the TGA thermogram intersects with the baseline drawn at the point where the descension of the curve occurs. The temperature for 5% weight loss is the temperature where 5 wt % of the starting material is thermally degraded.

### 2.3 Synthesis of Monomers

**Scheme 1:**
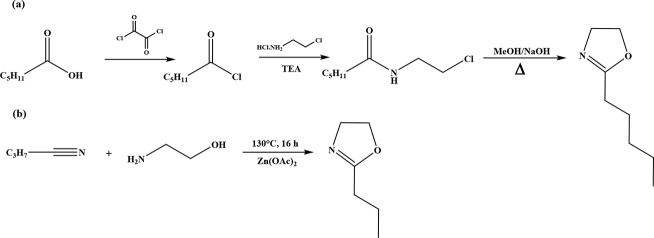
Synthesis of 2-Oxazoline Monomers: (a) PeOZ and (b) PrOZ Synthesis Starting from (a) Carboxylic acids and (b) Nitriles, Respectively

2-Oxazoline monomers are mainly synthesized via two main routes: (1) a one-step synthesis method starting from nitrile groups^41^ and (2) a three-step synthesis method starting from carboxylic acids.^5,42^ In this study, the nitrile route was employed to synthesize PrOZ monomer while PeOZ monomer was synthesized using 1-hexanoic acid as the precursor. The 2-oxazoline monomer synthesis methods used are shown in Scheme 1.

#### 2.3.1 Synthesis of 2-Propyl-2-oxazoline

Synthesis of PrOZ was carried out using a method reported in the literature with slight modifications.^43^ Butyronitrile (60 g, 0.87 mol, 1 equiv) was mixed with ethanol amine (58.5 g, 0.95 mol, 1.1 equiv) and a catalytic amount of Zn(OAc)_2_·2H_2_O (3.82 g, 0.017 mol, 0.02 equiv). The flask was heated to 130 °C under reflux and kept stirring at this temperature for 16 h. The reaction mixture turned to light brown when cooled down to room temperature. After cooling to room temperature, 200 mL of DCM was added to the mixture. The mixture was washed twice with deionized (DI) water and once with brine solution. The organic phase was separated and dried over Na_2_SO_4_ (14 g). This step was followed by the filtration of Na_2_SO_4_. DCM present in the filtrate was evaporated under reduced pressure, and the residue was distilled to obtain 52 g of colorless liquid monomer (yield: 53%), which was kept over 4 Å molecular sieves.

#### 2.3.2 Synthesis of 2-Pentyl-2-oxazoline

##### 2.3.2.1 Synthesis of Hexanoyl Chloride

Oxalyl chloride (82 g, 0.64 mol, 1.5 equiv) was added dropwise to ice-cooled hexanoic acid (50 g, 0.43 mol, 1 equiv) under N_2_ purge. The reaction mixture was allowed to warm to room temperature and then was heated to reflux for 1 h. After cooling down to room temperature, the excess oxalyl chloride was removed by distillation at 61 °C under 1 atm pressure. The product (hexanoyl chloride) was used in the next step without further purification.

##### 2.3.2.2 Synthesis of *N*-(2-Chloroethyl) Hexanamide

Triethylamine (161.8 mL, 1.161 mol, 2.7 equiv) was added to 700 mL of ice-cooled DCM in a 1 L round-bottom flask followed by the addition of 2-chloroethylamine hydrochloride (62.3 g, 0.537 mol, 1.25 equiv) into the flask. After purging with N_2_, the hexanoyl chloride obtained in the first step was added to the ice-cooled flask, dropwise. The mixture was allowed to warm to room temperature and kept stirring at room temperature overnight. The mixture was filtered to remove triethylamine hydrochloride, and the filtrate was evaporated under vacuum to remove DCM and excess TEA. DCM was then added to dissolve the product. The solution was washed twice with sodium bicarbonate solution and once with brine. The organic phase was separated and dried over Na_2_SO_4_ followed by its filtration. DCM present in the filtrate was evaporated under reduced pressure to yield 55.7 g of *N*-(2-chloroethyl) hexanamide as a solid product at room temperature (yield: 73%).

##### 2.3.2.3 Synthesis of 2-Pentyl-2-oxazoline Monomer (PeOZ)

The *N*-(2-chloroethyl) hexanamide (55.7 g, 1 equiv) obtained in the second step was dissolved in methanol (550 mL) to yield an overall concentration of 10 wt % in methanol and added into a 180 mL methanolic solution of NaOH (18,8 g of NaOH, 1.5 equiv). The solution was heated to reflux and kept stirring overnight. After cooling down to room temperature, the mixture was filtered and the filtrate was concentrated under reduced pressure. The solution was diluted with 40 mL of distilled water and extracted into DCM (3× 150 mL). Organic phases were combined and dried with Na_2_SO_4_ (30 g). Na_2_SO_4_ was filtered, and DCM present in the filtrate was evaporated under reduced pressure. The residue was distilled at 38 °C under vacuum (at 1 mbar) to obtain 24.0 g of 2-pentyl-2-oxazoline (yield: 54%).

### 2.4 General Copolymer Synthesis Procedure

**Scheme 2:**
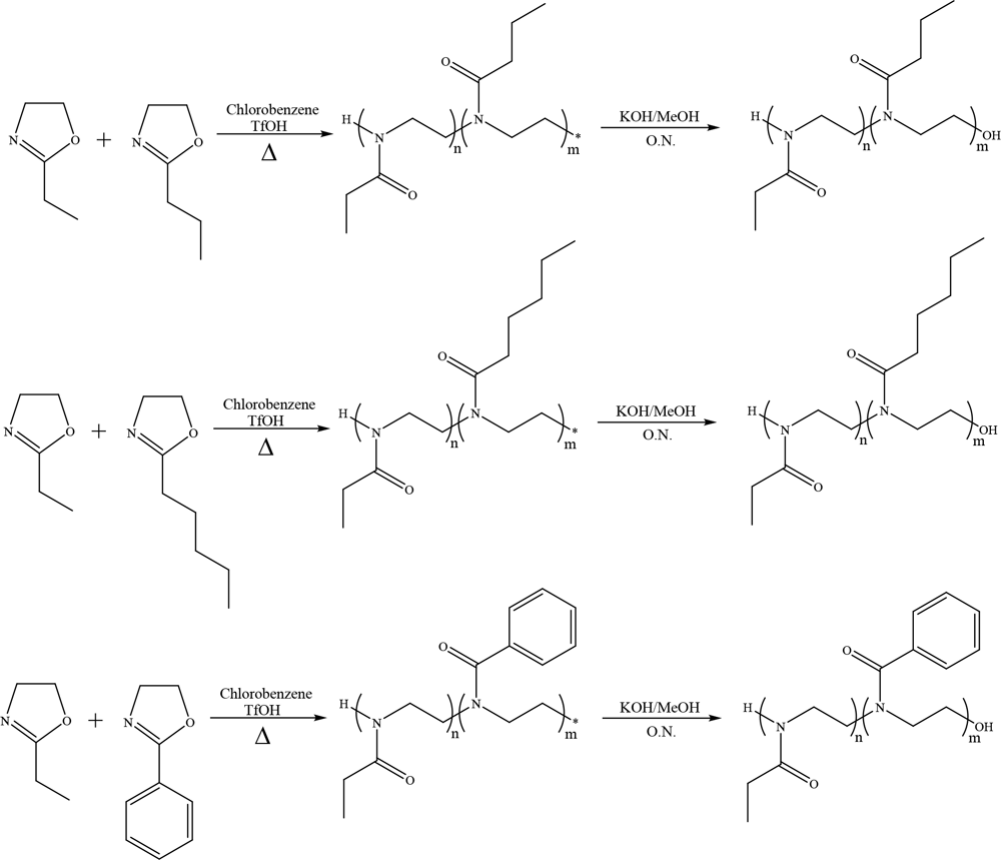
Synthesis of the Copolymers *m*:*n* ratios are 25:75, 50:50, and 75:25.

Polymerizations were carried out in round-bottom flasks under continuous N_2_ purge. Before introducing materials, the flasks were heated to 120 °C, vacuumed, and cooled to ambient temperature under nitrogen flow. Polymerizations were performed in chlorobenzene with an overall monomer concentration of 4 M. TfOH was used as the polymerization initiator. The monomer to initiator ratio ([M]/[I]) was set to be 10, 20, and 50 to obtain different degrees of polymerization and molar masses of 1000 g mol^–1^, 2000 g mol^–1^, and 5000 g mol^–1^, respectively. The polymerization times and temperatures for the copolymers are presented in Table 1. After cooling the reaction mixture to room temperature using an ice bath, polymerizations were terminated with methanolic KOH solution overnight.^44^ To investigate the effect of varying the copolymer composition on its properties, a number of random copolymers were synthesized through the copolymerization of EOZ with PrOZ, PeOZ, or PhOZ with the targeted molecular weights of 1000, 2000, and 5000 g mol^–1^ following the general polymerization procedure described above. The [M]/[I] was calculated by taking into account the total concentrations of two monomers. The targeted compositions were 25:75, 50:50, and 75:25 (molar ratio) for PEOZ:other polymer (PPrOZ, PPeOZ, PPhOZ) resulting in 27 copolymers. The synthesis of the copolymers is shown in Scheme 2.

**Table 1:** Polymerization Conditions for Copolymers

	Temp (°C)	Time (min)
PEOZ–PPrOZ	80	30, 60, and 120 for 1, 2, and 5 kg mol^–1^, respectively
PEOZ–PPeOZ	80	60, 120, and 240 for 1, 2, and 5 kg mol^–1^, respectively
PEOZ–PPhOZ	95	60, 120, and 240 for 1, 2, and 5 kg mol^–1^, respectively

#### 2.4.1 Purification of PEOZ–PPrOZ and PEOZ–PPeOZ

The solvents of the reaction mixture (chlorobenzene and methanol) were evaporated under reduced pressure. DCM was added to the resulting white solid to dissolve the polymer. The mixture was filtered to remove the excess KOH, and the filtrate was dried with Na_2_SO_4_ followed by its filtration and evaporation of the solvent under reduced pressure. The polymer was dissolved in methanol and precipitated into ice-cooled diethyl ether. The precipitate was filtered and dried under vacuum at room temperature to give the final polymers. This purification procedure was followed for the purification of PEOZ–PPrOZ and PEOZ–PPeOZ copolymers having different compositions and molecular weights.

#### 2.4.2 Purification of PEOZ–PPhOZ

The solvents (chlorobenzene and methanol) of the reaction mixture were evaporated under reduced pressure. THF was added to the resulting white solid to dissolve the copolymer. The mixture was filtered to remove the excess KOH, and the filtrate was evaporated under reduced pressure. The copolymer was dissolved in methanol and precipitated into ice-cooled diethyl ether. The precipitate was filtered and dried at room temperature under vacuum to give the final copolymers. This purification procedure was followed for the purification of PEOZ–PPhOZ copolymers with different compositions and molecular weights.

## 3 Results and Discussion

### 3.1 Structural Analysis of Synthesized and Commercial Monomers

**Figure 1:**
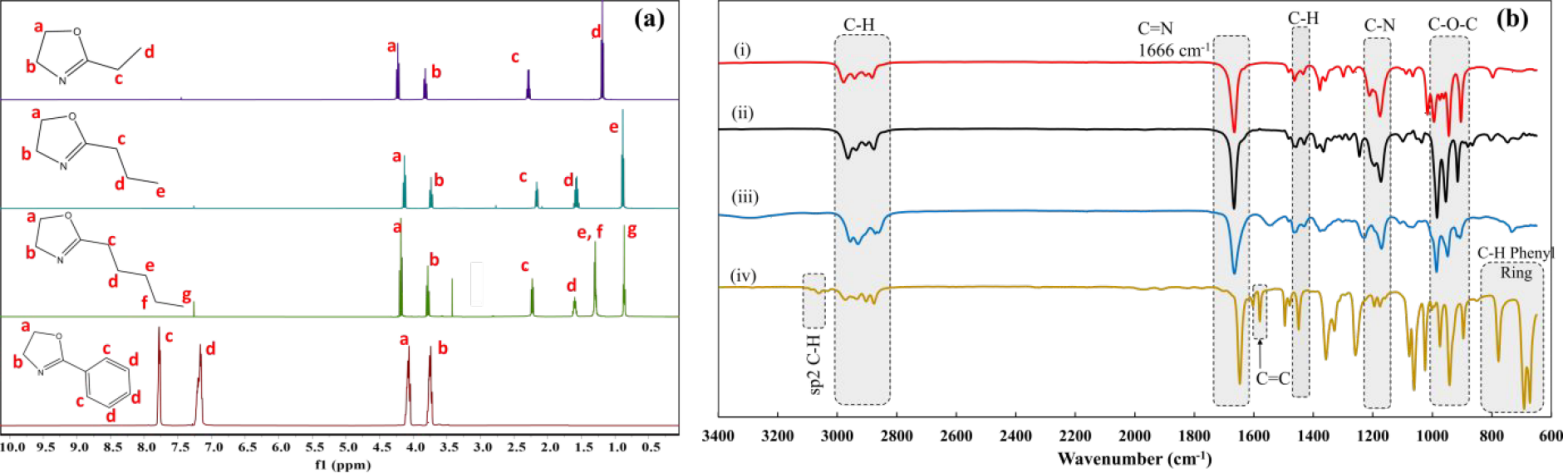
(a) ^1^H NMR spectra of EOZ, PrOZ, PeOZ, and PhOZ recorded in CDCl_3_ and (b) FTIR spectra of 2-oxazoline monomers: (a) EOZ, (b) PrOZ, (c) PeOZ, and (d) PhOZ.

Structural analysis of the synthesized (PrOZ and PeOZ) and commercial (EOZ and PhOZ) monomers was performed by ^1^H NMR and FTIR as shown in Figure 1a,b, respectively. On the ^1^H NMR spectra, two peaks associated with protons of the oxazoline ring (a and b) appeared at δ 3.75 and δ 4.10 ppm. The transformation of these peaks to new peaks associated with the protons of the polymer backbone was monitored in order to follow the polymerizations. The peaks associated with the protons present on the side groups are assigned on the spectra with matching integral values to the number of hydrogen atoms of methylene and methyl groups. In the FTIR spectra shown in Figure 1b, the sharp peak at 1660 cm^–1^ is attributed to the stretching of C=N of the heterocyclic ring in all monomers.^45^ For the PhOZ, the C=C peak is marked at 1570 cm^–1^. For all of the monomers, asymmetric and symmetric stretches of C–O–C peaks appeared at around 1000 cm^–1^ in the fingerprint region. The peaks associated with C–H bonds (CH bonds in CH_2_ and CH_3_ groups) on the 2-oxazoline ring and the side groups appeared at 1470 cm^–1^ and around 3000 cm^–1^, respectively. The peak associated with sp^2^ hybridized C–H of the phenyl ring appeared at 3100 cm^–1^. Out of plane bending of C–H on phenyl ring can be related to the peaks that appeared between 600 and 800 cm^–1^.^46^ The characteristic peaks are provided on the spectra that confirm the structure of synthesized and commercial monomers. To follow the polymerizations, the transformations of C–O and C=N peaks of the monomers were monitored during polymerizations.

### 3.2 Structural Analysis of Synthesized Copolymers

**Figure 2:**
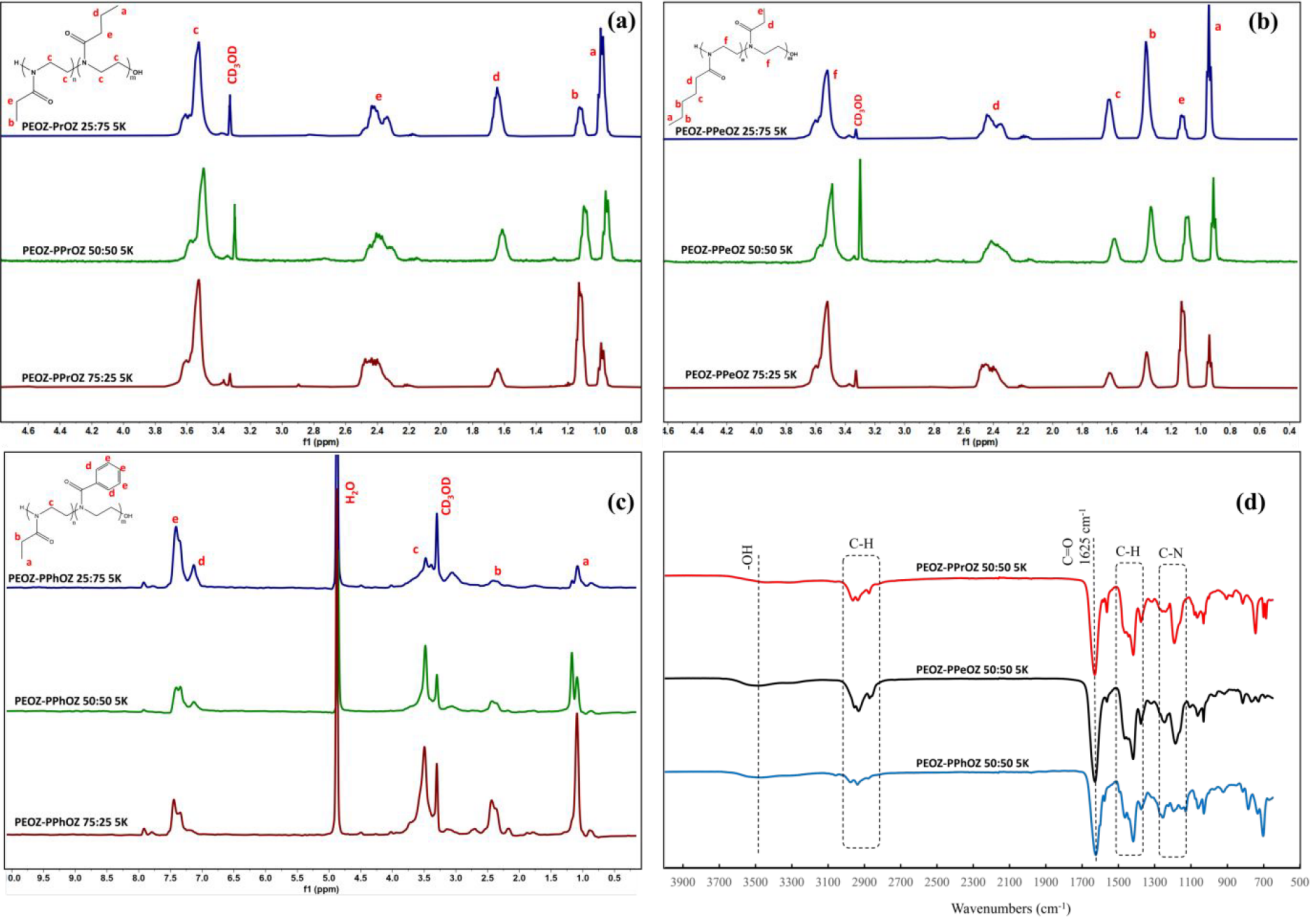
Structural analysis of copolymers. ^1^H NMR spectra of (a) PEOZ–PPrOZ 5K, (b) PEOZ–PPeOZ 5K, and (c) PEOZ–PPhOZ 5K at different compositions. (d) FTIR spectra of copolymers with different side groups at the same composition.

To thoroughly investigate the effects of the systematic variation of molecular weight and composition on the properties of the copolymer, a series of random POZ copolymers with targeted molar masses of 1000, 2000, and 5000 g mol^–1^ and compositions of 25:75, 50:50, and 75:25 were synthesized. All polymerizations were initiated with TfOH and terminated with methanolic KOH solution. The synthesized copolymers were analyzed by ^1^H NMR and FTIR to confirm their structures. Figure 2a–c shows the ^1^H NMR spectra of PEOZ–PPrOZ 5K, PEOZ–PPeOZ 5K, and PEOZ–PPhOZ 5K random copolymers with different compositions, respectively. The ^1^H NMR spectra of copolymers show the disappearance of two peaks at δ 3.75 and δ 4.10 ppm, which are assigned to the protons of the 2-oxazoline monomer ring. The peaks associated with the protons of the methylene groups on the polymer backbone appeared at δ 3.4–3.6 ppm. The ^1^H NMR results were used to determine the final compositions of copolymers. To compare the composition of synthesized copolymers with the feed ratio of monomers, the distinct peaks of the methyl groups of the alkyl or C–H peaks of aryl side chains on ^1^H NMR spectra were integrated. The increases in the intensity of the peaks associated with the protons of the methyl groups of the side chains of PEOZ (peak “b”) compared to the protons of the methyl groups of the side chains of PPrOZ and PPeOZ (peak “d”) were clearly observed from the spectra as the ratios of the ethyl group to the propyl or pentyl group were varied from 25:75, to 50:50, and then to 75:25. To calculate the composition of the PEOZ–PPhOZ copolymer, the peaks associated with the protons of the phenyl group (δ 7.4 ppm) were compared to the protons of the methyl group of the side chain of PEOZ (δ 1.1 ppm). Table 2 summarizes the compositions of the obtained copolymers and the monomer feed ratios. The results indicate that the copolymer compositions are the same as the monomer feed ratios, which show that, at the implemented polymerization conditions, the ratio of the monomer feed determines the final composition of the copolymers. This behavior demonstrates the proximity of the polymerization rate of used monomers at given temperatures.

**Table 2:** Molecular Weight Results of Copolymers (by SEC) and Copolymer Composition Results (by ^1^H NMR) along with Reaction Yields

		SEC						
		PEOZ std			PMMA std			
Copolymer	Composition by ^1^H NMR	*M*_p_ (Da)	*M*_n_ (Da)	*Đ*	*M*_p_ (Da)	*M*_n_ (Da)	*Đ*	yield (%)
PEOZ–PPrOZ 25:75 1K	25:75	794	650	1.18	1594	917	1.61	74
PEOZ–PPrOZ 25:75 2K	25:75	1642	1,240	1.28	3452	2309	1.4	73
PEOZ–PPrOZ 25:75 5K	26:74	4117	3,643	1.21	7863	7085	1.15	76
PEOZ–PPrOZ 50:50 1K	43:57	752	626	1.15	1206	738	1.5	72
PEOZ–PPrOZ 50:50 2K	51:49	1702	1,338	1.2	2870	2130	1.24	76
PEOZ–PPrOZ 50:50 5K	50:50	5203	3,606	1.3	8291	6032	1.23	77
PEOZ–PPrOZ 75:25 1K	72:28	678	535	1.18	1305	591	1.79	77
PEOZ–PPrOZ 75:25 2K	75:25	1778	1552	1.15	3726	3214	1.14	76
PEOZ–PPrOZ 75:25 5K	74:26	4746	3697	1.2	7666	6173	1.16	81
PEOZ–PPeOZ 25:75 1K	25:75	1000	854	1.13	2076	1634	1.2	69
PEOZ–PPeOZ 25:75 2K	23:77	1909	1503	1.23	3981	2923	1.3	71
PEOZ–PPeOZ 25:75 5K	25:75	4915	2910	1.46	9134	5739	1.36	73
PEOZ–PPeOZ 50:50 1K	49:51	964	812	1.20	1956	1532	1.25	70
PEOZ–PPeOZ 50:50 2K	52:48	1823	1662	1.18	3812	3433	1.17	71
PEOZ–PPeOZ 50:50 5K	51:49	4784	4218	1.36	8930	8076	1.26	74
PEOZ–PPeOZ 75:25 1K	73:27	925	715	1.21	1903	1083	1.58	73
PEOZ–PPeOZ 75:25 2K	75:25	1937	1665	1.15	4034	3446	1.14	76
PEOZ–PPeOZ 75:25 5K	74:26	5251	3782	1.3	9657	7335	1.22	78
PEOZ–PPhOZ 25:75 1K	30:70	1189	832	1.331	2501	2237	1.24	73
PEOZ–PPhOZ 25:75 2K	27:73	2175	1681	1.19	4489	3461	1.18	75
PEOZ–PPhOZ 25:75 5K	22:78	4622	3327	1.29	8647	6527	1.22	76
PEOZ–PPhOZ 50:50 1K	49:51	1035	1057	1.127	2157	2156	1.14	74
PEOZ–PPhOZ 50:50 2K	52:48	2332	1815	1.2	4783	3733	1.18	75
PEOZ–PPhOZ 50:50 5K	46:54	4999	3690	1.29	8798	6954	1.20	77
PEOZ–PPhOZ 75:25 1K	75:25	863	1002	1.13	1759	2033	1.15	76
PEOZ–PPhOZ 75:25 2K	76:24	1871	1538	1.19	3905	3181	1.18	77
PEOZ–PPhOZ 75:25 5K	74:26	3596	2288	1.39	7005	4614	1.32	79

The synthesized copolymers were also characterized by FTIR spectroscopy to confirm their structures. Figure 2d shows the FTIR spectra of copolymers with 5000 g mol^–1^ molar mass and 50:50 composition. The sharp peak associated with C=N stretching of monomers at 1640 cm^–1^ is altered to an amide peak present in the polymer structure at 1660 cm^–1^. The peaks associated with the C–O stretching of the monomer ring disappeared after the polymerizations. Out of plane bending peaks of the phenyl ring are observed in the range 600–800 cm^–1^ along with the C=C peak of phenyl ring at 1579 cm^–1^.^47^ The −OH stretching peak appeared on the copolymer spectra, which was not visible on dry monomers. This peak can be associated with the terminal −OH group and the absorbed moisture resulting from the hydrophilicity of PEOZ carrying copolymers. FTIR spectra of the copolymers with targeted molar masses of 1000 and 2000 g mol^–1^ and different compositions exhibited similar peaks as the copolymers with molar mass of 5000 g mol^–1^ and 50:50 composition.

The molecular weight and dispersity values of the synthesized copolymers measured by SEC are shown in Table 2. Moreover, values for yields are reported in Table 2 based on the amount of the material obtained after the purification steps.

The SEC measurements were performed using two different standards of poly(methyl methacrylate) (PMMA) and the prepared PEOZ homopolymers standard. Since the synthesized copolymers have an amphiphilic nature, neither of these standards solely would have been sufficient to characterize the copolymers due to the hydrophilicity of PEOZ and hydrophobicity of PMMA. Thus, both standards were used to investigate the molar mass and *Đ* of the copolymers. Although the results do not show the exact molar mass, the range of the measurements indicated the successful synthesis of copolymers with molar masses of 1000, 2000, and 5000 g mol^–1^. Moreover, the proximity of the molar masses of different copolymers having the same targeted molecular weight in both standards indicated the accuracy of making a comparison among them to investigate the effect of other parameters on polymer properties other than the molecular weight. As presented in Table 2, synthesized copolymers have narrow polydispersity values which confirm the living nature of the polymerization.

### 3.3 Thermal Analysis

#### 3.3.1 DSC Results

**Figure 3:**
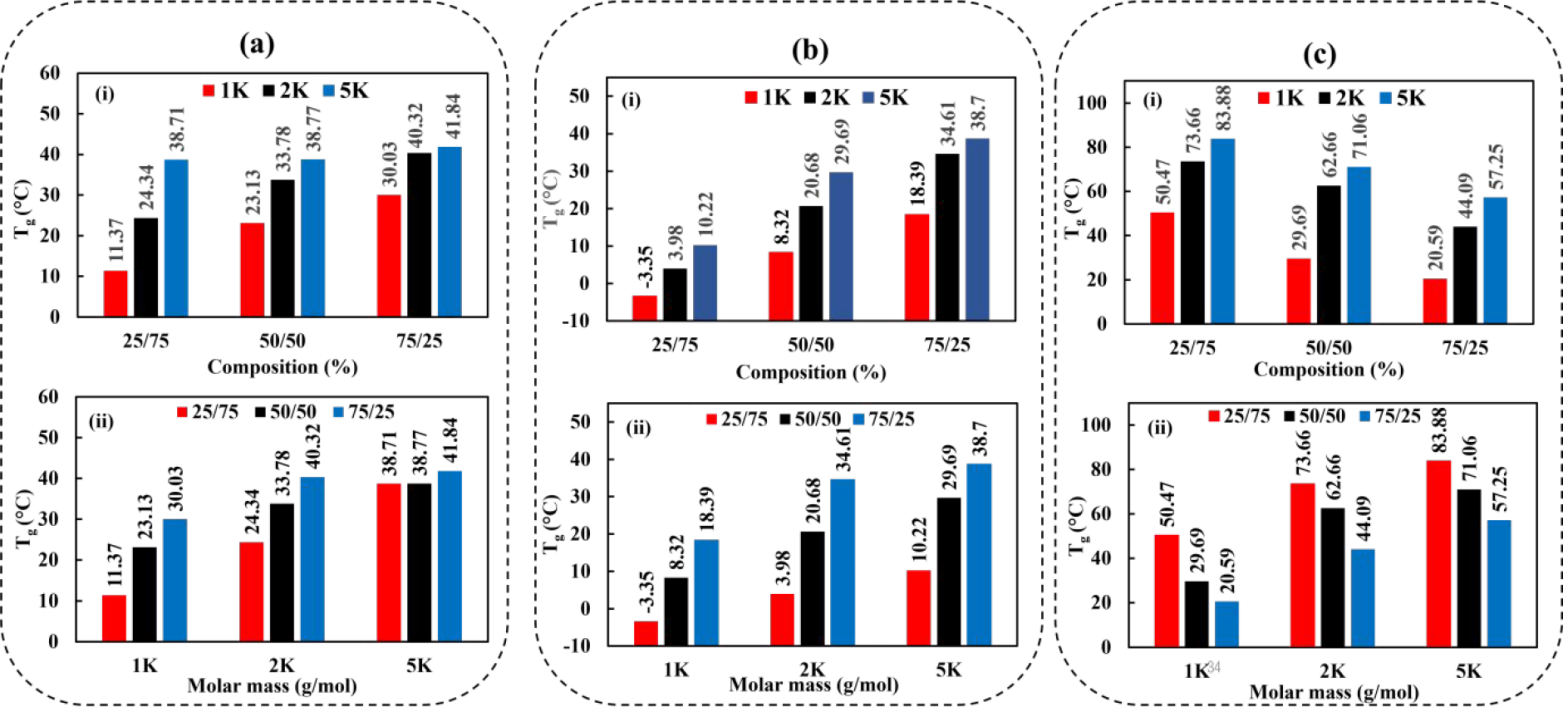
*T*_g_ values of copolymers based on composition (i) and molar mass (ii): (a) PEOZ–PPrOZ, (b) PEOZ–PPeOZ, and (c) PEOZ–PPhOZ.

The thermal properties such as *T*_g_ and crystallinity of the synthesized copolymers were studied using DSC. As reported in the literature, the length of the polymer side chain and the number of repeating units act as influential factors in determining the crystallinity and *T*_g_ of the polymer.^48^ The effects of having aliphatic chains with two, three, and five carbons and phenyl group as the aromatic group on the thermal properties of the copolymers of different molecular weights and compositions have been studied. Figure 3 shows the *T*_g_ values of PEOZ–PPrOZ, PEOZ–PPeOZ, and PEOZ–PPhOZ copolymers based on composition and molar mass. DSC thermograms are presented in Supporting Information Figures S1–S3. The copolymerization of EOZ with PrOZ, PeOZ, and PhOZ has resulted in copolymers having a *T*_g_ value that lies in between the *T*_g_ values of the homopolymers of each monomer depending on the copolymer composition. The presence of only one *T*_g_ without *T*_c_ and *T*_m_ for all synthesized copolymers indicated their amorphous state and random structure.

As seen from Figure 3, copolymerization of a monomer having a longer aliphatic side chain than ethyl as in PEOZ has lowered the *T*_g_ of the obtained copolymers. The *T*_g_ values of PEOZ–PPrOZ (Figure 3a,ii) and PEOZ–PPeOZ (Figure 3b,ii) copolymers at the same molecular weights increased as the molar content of EOZ increased from 25% to 50% and to 75% in the copolymer composition. This can be attributed to having a higher content of short side chains resulting in higher packing density and stronger amide dipole interactions arising from the proximity of the backbones of the copolymer chains. Unlike the homopolymers of PPeOZ that are in a semicrystalline state, only a *T*_g_ value is observed for the synthesized PEOZ–PPeOZ copolymers. The incorporation of PEOZ, even at a low molar ratio (e.g., 25%), in the copolymer structure with PPeOZ causes the neighboring 2-pentyl side chains to be pushed apart preventing the side chain crystallinity. Thus, with the decreased packing density and in the absence of side chain crystallinity, the semicrystalline structure of PPeOZ homopolymer is altered to an amorphous state for the PEOZ–PPeOZ copolymers. Investigating the *T*_g_ values of the copolymers of the same molar mass having aliphatic side chains reveals the effect of the length of the side chain on the copolymer properties. The *T*_g_ value of the copolymer is decreased with the increase in the content of the monomer with a longer side chain. For instance, as shown in Figure 3a,ii, *T*_g_ values for PEOZ–PPrOZ 1K copolymers are 11.37, 23.13, and 30.03 °C while for PEOZ–PPeOZ 1K copolymers, *T*_g_ values are −3.35, 8.32, and 18.39 °C, for the compositions of 25:75, 50:50, and 75:25, respectively. The decrease in *T*_g_ values has resulted in obtaining rubber-like materials with a *T*_g_ lower than ambient temperature. The rubbery characteristic of the obtained copolymers can be exploited for its plasticizing effects by having long and hydrophobic alkyl side chains while avoiding the crystallinity resulting from incorporating PEOZ in the polymer structure.

For PEOZ–PPhOZ copolymers having the same molar mass, an increasing trend was observed for *T*_g_ values with the increase in the content of PhOZ or decrease in the content of EOZ. The increase in *T*_g_ with respect to the increase in PhOZ content is attributed to the presence of rigid aromatic phenyl ring and possible π-stacking of phenyl side groups.

Investigating the effects of altering molar mass on *T*_g_ revealed that an increase in the molar mass had resulted in higher *T*_g_ values for all copolymers of the same composition. For all copolymers, as the molar mass increased, a higher *T*_g_ value was observed due to the stronger interactions of the copolymer chains having a higher number of repeating units. Moreover, the higher number of repeating units resulted in more entanglements of the polymer chains which resulted in higher *T*_g_ values.

The DSC results revealed that the length, structure, and interactions (functionalities) of side chains along with the molar mass of the copolymer affect the thermal properties of the copolymers such as *T*_g_. With a longer side chain, the *T*_g_ is lowered since the longer side chain lowers the packing density and facilitates the chain motion. Moreover, having a shorter side chain results in the proximity of backbones of the copolymer chains. Thus, the amide dipole interactions are stronger leading to a higher *T*_g_. On the other hand, longer side chains increase the distance between amide groups of polymer chains, which facilitates the relaxation of the backbone of copolymer chains, thus decreasing the *T*_g_.^49^ For the copolymers with aromatic side chains, interactions of the side chain functionalities were the dominant factor in determining the thermal properties. The π-stacking of 2-phenyl substituents possibly increased the packing density of the polymer chains, which resulted in higher *T*_g_ values. As all the copolymers show a single *T*_g_ value without crystallization or melting peaks, it can be stated that all copolymers are in an amorphous and random state.

**Figure 4:**
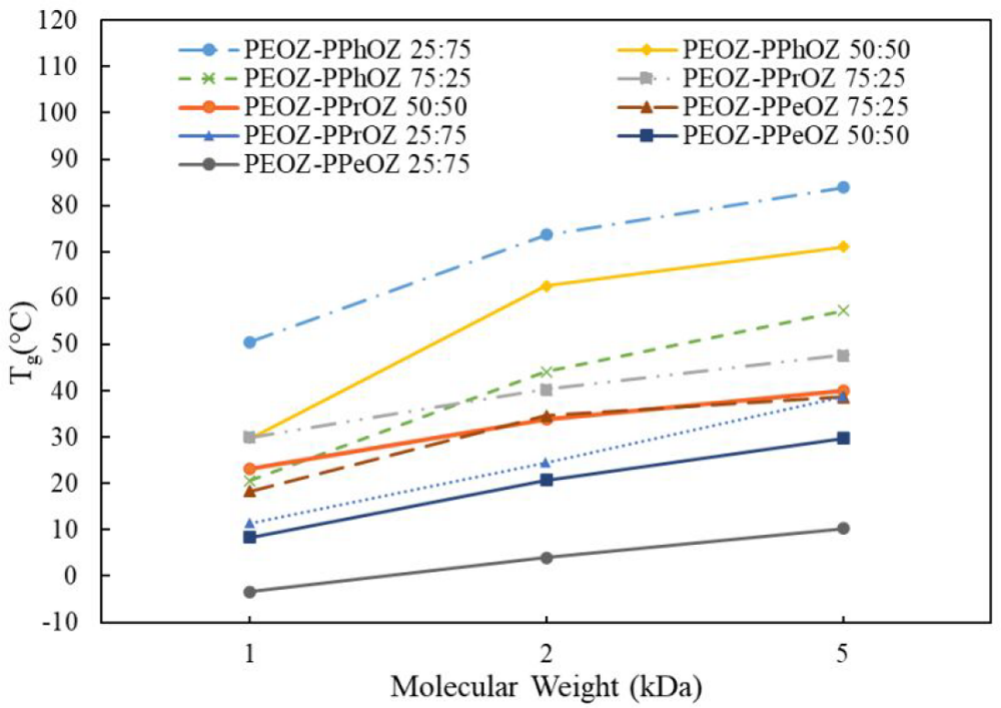
Comparison of *T*_g_ values of the copolymers with different molecular weights.

Figure 4 shows the *T*_g_ values of the synthesized copolymers with respect to their molecular weight. For each copolymer, *T*_g_ increased with an increase in the molecular weight of the polymer. For all molecular weights, it was observed that the PEOZ–PPeOZ 25:75 copolymer has the lowest *T*_g_ value due to having the highest number of the longest alkyl side chains among the copolymers. Additionally, for all molecular weights, PEOZ–PPhOZ 25:75 copolymer has the highest *T*_g_ value due to the presence of the highest content of the 2-phenyl substituent groups on the polymer structure. It is noticeable that, for a desired *T*_g_ value (e.g., 34 °C), multiple copolymers with different side chains and molecular weights (i.e., PEOZ–PPeOZ75:25 2K, PEOZ–PPrOZ 50:50 2K, and PEOZ–PPrOZ 25:75 5K) can be synthesized and utilized based on the required final properties. This multiplicity of choices provides the opportunity to obtain the required final properties (e.g., *T*_g_) while having the possibility to manipulate other characteristics of the polymer such as molecular weight, amphiphilicity, solution viscosity, and functionalities.

#### 3.3.2 TGA Results

Thermal stability and degradation temperatures of the copolymers were determined by TGA. The results indicate that the thermal degradation of POZ copolymers with aliphatic side chains proceeds with one-step decomposition. However, the incorporation of the 2-phenyl substituent in the polymer structure gives rise to further decomposition shoulders, which results in a multistep decomposition. This behavior is observed for all compositions of PEOZ–PPhOZ copolymers. The multistep shoulders indicate that PEOZ–PPhOZ copolymers have more complex decomposition mechanisms compared to the copolymers with aliphatic chains.^50^

**Figure 5:**
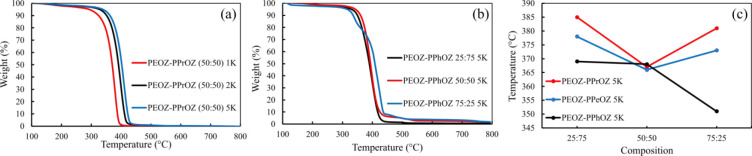
TGA thermograms of PEOZ–PPrOZ copolymers with different molecular weights (a) and PEOZ–PPhOZ having different compositions (b). Comparison of degradation temperatures for different compositions of the copolymers with a molecular weight of 5 kDa (c).

**Figure 6:**
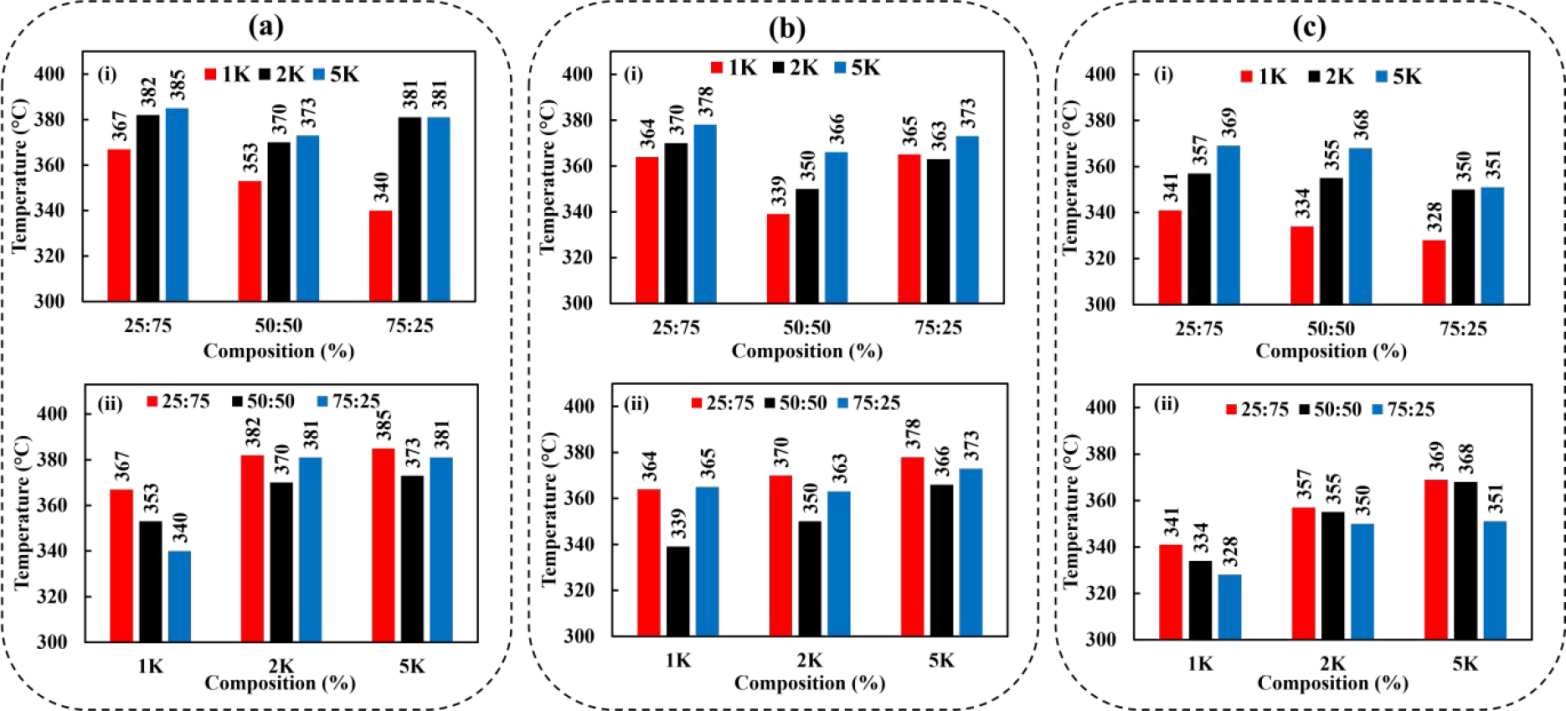
Onset temperatures of thermal degradation for copolymers based on composition (i) and molar mass (ii): (a) PEOZ–PPrOZ, (b) PEOZ–PPeOZ, and (c) PEOZ–PPhOZ.

In general, the weight loss seen below 120 °C is related to the evaporation of water absorbed by the copolymers. The copolymer decomposition starts above 300 °C, and the onset temperatures of copolymers are in the range 328–383 °C. During degradation steps, the weight loss is completed with less than 5% residue, which validates the efficiency of the implemented purification methods to remove the excess KOH used for termination. Figure 6 shows the onset temperature of degradation for copolymers based on composition and molar mass. The decomposition temperatures demonstrate a decreasing trend with respect to the increase in the length of the aliphatic side chain. When the decomposition temperatures of all copolymers of the same compositions are investigated, they increase with respect to the increasing molecular weight. For the polymers with aliphatic side chains having the same molecular weight, the copolymers with a composition closer to the homopolymer of each type (i.e., 25:75 and 75:25) have higher decomposition temperatures compared to the composition of 50:50 that has lower decomposition temperatures. For copolymers of the same molecular weight having a phenyl group as the side chain, the decomposition temperatures increase with the increase in the PhOZ content. This behavior is also shown in Figure 5c where decomposition temperatures of PEOZ–PPrOZ 5K, PEOZ–PPeOZ 5K, and PEOZ–PPhOZ 5K copolymers are examined.The degradation parameters, such as onset temperature (*T*_d,onset_), completion temperature (*T*_d,endset_), and 5% weight loss (*T*_d,5%_) are summarized in Table S1 (see SI).

## 4 Conclusions

A series of POZ copolymers were synthesized and characterized to investigate the structure–property relationship by altering the side chain, composition, and molecular weights of the copolymers. The copolymers were synthesized from copolymerization of PrOZ, PeOZ, and PhOZ with EOZ in three different compositions of 25:75, 50:50, and 75:25. The targeted molecular weights of the copolymers were 1000, 2000, and 5000 Da. FTIR and ^1^H NMR results confirm the successful synthesis of the monomers and copolymers with the desired compositions. SEC measurements indicate a narrow polydispersity, which confirms the living nature of the cationic ring-opening polymerization. The thermal properties of the copolymers were studied using DSC and TGA. DSC results revealed that copolymers are amorphous and random have a single *T*_g_ and do not show any crystallization or melting peaks. By altering the side chain of the copolymers along with having different molecular weights, a wide range of *T*_g_ values from −3 to 84 °C is obtained, which provides the possibility to have the desired *T*_g_ value with the favored molecular weight and side chain. It was observed that increasing the length of the alkyl side chain resulted in a decrease in *T*_g_ values because of the lower packing density while the incorporation of 2-phenyl substituents resulted in an increase in *T*_g_ values due to the π-stacking. Moreover, regardless of the side chain, *T*_g_ increased with the increase in the molecular weight of the copolymers. The DSC results revealed the effect of the number of repeating units on the packing density and the number of entanglements of the copolymers. TGA results indicated a single-step degradation for copolymers having aliphatic side chains while the copolymers carrying 2-phenyl substituent groups exhibited a multistep degradation revealing a more complex degradation mechanism for these copolymers. It was also observed that the longer aliphatic side chain results in a lower degradation temperature. Moreover, the degradation temperatures have an increasing trend with an increase in the molecular weights of the copolymers. The effect of the copolymer composition on the degradation temperature was also studied. It was observed that, for the copolymers having aliphatic side chains, the 50:50 composition possesses the lowest degradation temperature compared to other compositions. For the PEOZ–PPhOZ copolymers, increasing the PhOZ content resulted in an increase in the degradation temperature.

## Supporting Information Available

DSC thermograms and degradation temperatures (PDF)
