# Supplementary material for: Synthesis and Structure–Property Relationship of Amphiphilic Poly(2-ethyl-co-2-(alkyl/aryl)-2-oxazoline) Copolymers
Source: ACS Omega. 2022 Oct 26;7(44):40067–77. doi: 10.1021/acsomega.2c04809 (PMC9648074; doi:10.1021/acsomega.2c04809)
Supplement: Supplementary file 1 — ao2c04809_si_001.pdf [file ao2c04809_si_001.pdf]

Supporting Information

Synthesis and Structure-Property Relationship of Amphiphilic Poly (2-Ethyl-co-2-(Alkyl/Aryl)-2-Oxazoline) Copolymers

Taha Behroozi Kohlan[1, 2], Asu Ece Atespare[1, 2], Mehmet Yildiz[1,2], Yusuf Ziya Menceloglu[1, 2], Serkan Unal[1, 2], Bekir Dizman[1, 2] *

[1] Integrated Manufacturing Technologies Research and Application Center & Composite Technologies Center of Excellence, Sabanci University, Istanbul, Turkey

[2] Faculty of Engineering and Natural Sciences, Materials Science and Nano Engineering, Sabanci University, Istanbul, Turkey

*Corresponding author: Bekir Dizman

Index

Figure S1. DSC thermograms of PEOZ-PPrOZ copolymers

Figure S2. DSC thermograms of PEOZ-PPeOZ copolymers

Figure S3. DSC thermograms of PEOZ-PPhOZ copolymers

Table S1. Degradation temperatures of copolymers.

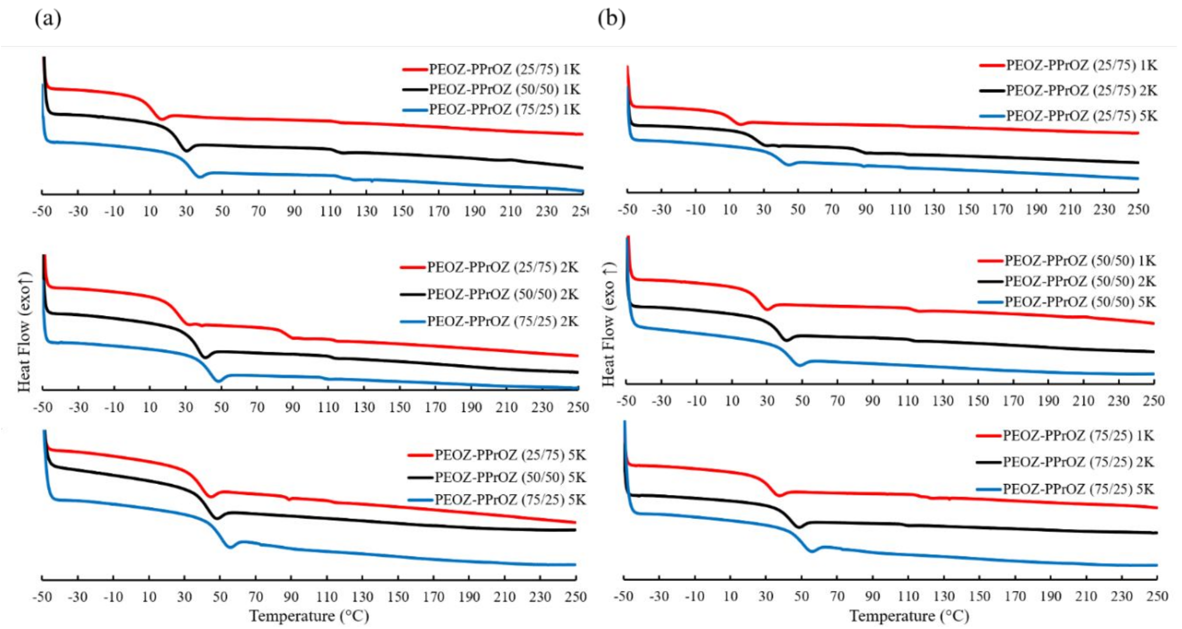

Figure S1. DSC thermograms of PEOZ-PPrOZ copolymers based on molecular weight (a) and composition (b).

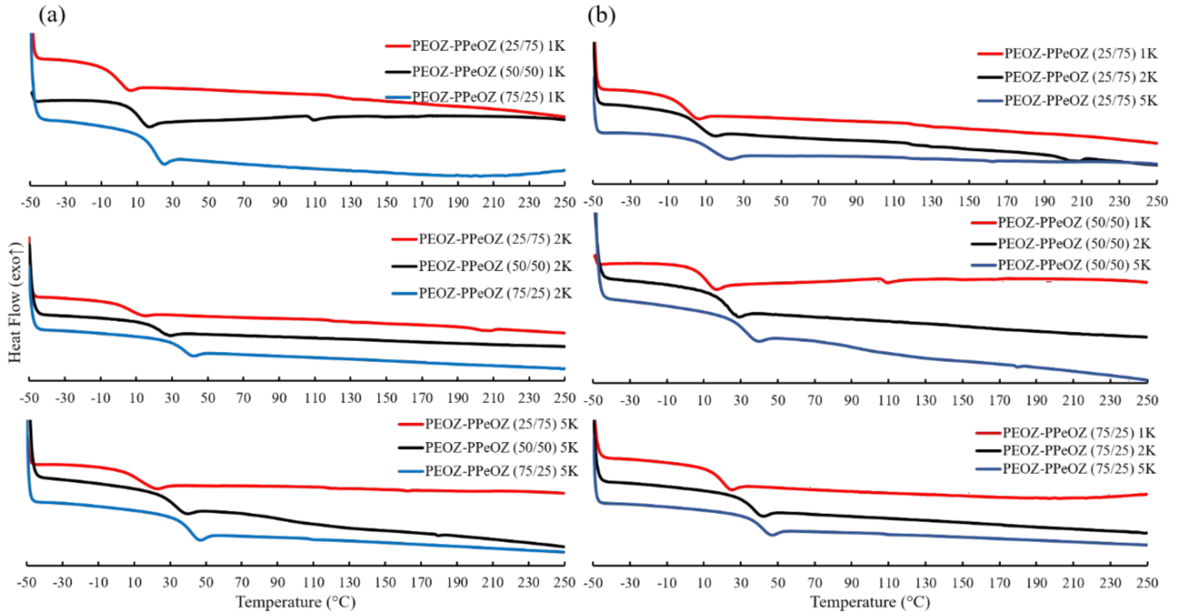

Figure S2. DSC thermograms of PEOZ-PPeOZ copolymers based on molecular weight (a) and composition (b).

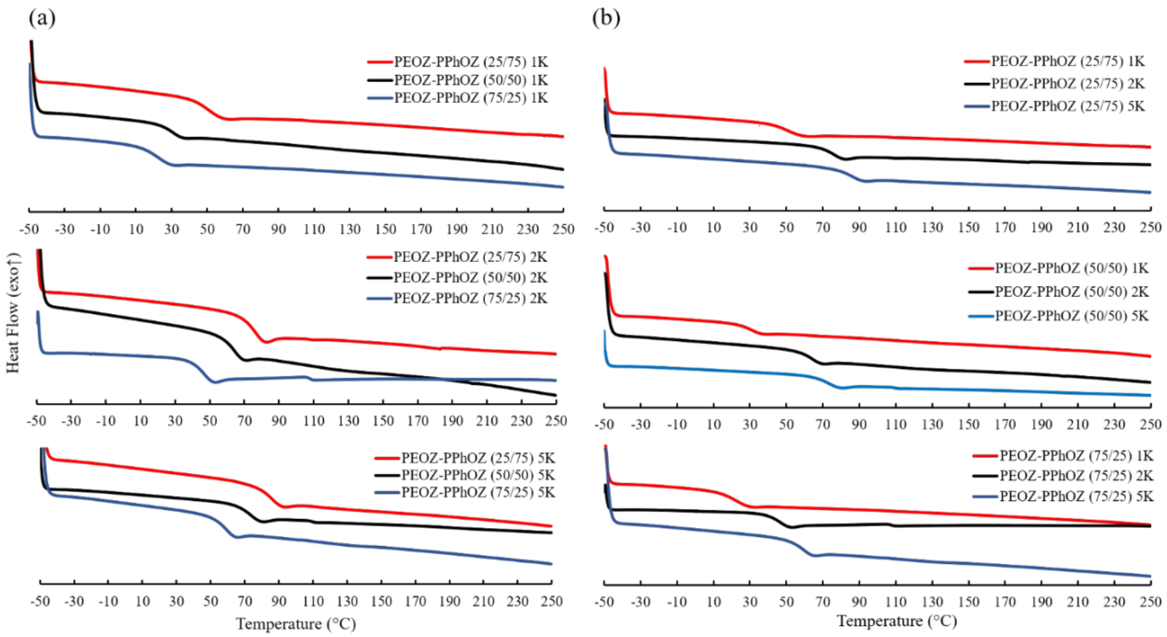

Figure S3. DSC thermograms of PEOZ-PPhOZ copolymers based on molecular weight (a) and composition (b).

Table S1. Degradation temperatures of copolymers.

Copolymers	T_d (°C) (onset)	T_d (°C) (endset)	T_d (5%) (°C)
PEOZ-PPrOZ 25:75 1K	367	418	320
PEOZ-PPrOZ 25:75 2K	382	428	340
PEOZ-PPrOZ 25:75 5K	385	419	370
PEOZ-PPrOZ 50:50 1K	353	391	319
PEOZ-PPrOZ 50:50 2K	370	414	346
PEOZ-PPrOZ 50:50 5K	373	404	361
PEOZ-PPrOZ 75:25 1K	340	378	302
PEOZ-PPrOZ 75:25 2K	381	421	358
PEOZ-PPrOZ 75:25 5K	381	427	365
PEOZ-PPeOZ 25:75 1K	364	415	314
PEOZ-PPeOZ 25:75 2K	370	413	348
PEOZ-PPeOZ 25:75 5K	378	416	351
PEOZ-PPeOZ 50:50 1K	339	396	357
PEOZ-PPeOZ 50:50 2K	350	401	324
PEOZ-PPeOZ 50:50 5K	366	408	349
PEOZ-PPeOZ 75:25 1K	365	421	309
PEOZ-PPeOZ 75:25 2K	363	405	335
PEOZ-PPeOZ 75:25 5K	373	419	352
PEOZ-PPhOZ 25:75 1K	341	420	308
PEOZ-PPhOZ 25:75 2K	357	429	320
PEOZ-PPhOZ 25:75 5K	369	474	333
PEOZ-PPhOZ 50:50 1K	334	462	313
PEOZ-PPhOZ 50:50 2K	355	467	317
PEOZ-PPhOZ 50:50 5K	368	407	353
PEOZ-PPhOZ 75:25 1K	328	378	313
PEOZ-PPhOZ 75:25 2K	350	396	330
PEOZ-PPhOZ 75:25 5K	351	465	304
